# Development of Tools for Integrated Monitoring and Assessment of Hazardous Substances and Their Biological Effects in the Baltic Sea

**DOI:** 10.1007/s13280-013-0478-3

**Published:** 2014-01-12

**Authors:** Kari K. Lehtonen, Brita Sundelin, Thomas Lang, Jakob Strand

**Affiliations:** 1 Marine Research Centre, Finnish Environment Institute, Hakuninmaantie 6, Helsinki, 00430 Finland; 2 Department of Applied Environmental Science, Stockholm University, Svante Arrhenius Väg 8, 114 18 Stockholm, Sweden; 3 Thünen Institute for Fishery Ecology, Deichstraße 12, 27472 Cuxhaven, Germany; 4 Department of Biosciences, Marine Ecology, Frederiksborgvej 399, 4000 Roskilde, Denmark

**Keywords:** Assessment, Baltic Sea, Biological effects, Biomarkers, Hazardous substances, Monitoring

## Abstract

The need to develop biological effects monitoring to facilitate a reliable assessment of hazardous substances has been emphasized in the Baltic Sea Action Plan of the Helsinki Commission. An integrated chemical–biological approach is vitally important for the understanding and proper assessment of anthropogenic pressures and their effects on the Baltic Sea. Such an approach is also necessary for prudent management aiming at safeguarding the sustainable use of ecosystem goods and Services. The BEAST project (Biological Effects of Anthropogenic Chemical Stress: Tools for the Assessment of Ecosystem Health) set out to address this topic within the BONUS Programme. BEAST generated a large amount of quality-assured data on several biological effects parameters (biomarkers) in various marine species in different sub-regions of the Baltic Sea. New indicators (biological response measurement methods) and management tools (integrated indices) with regard to the integrated monitoring approach were suggested.

## Introduction

Marine and coastal ecosystems around the globe are facing the threat of multiple anthropogenic activities causing severe impacts on their health. Among these, the effects of hazardous substances can be observed at all biological levels; consequently, their impacts must be considered when assessing the health status of marine ecosystems (Downs and Ambrose 2001). To increase the significance of impact assessments, there is now a need to bridge the gap between the measured concentrations of chemicals and their biological effects at different biological organization levels. Suitable tools and monitoring strategies have been developed and applied in various sea areas (Allen and Moore 2004; Broeg et al. 2005; Broeg and Lehtonen 2006; Moore et al. 2006; Dagnino et al. 2007; Strand 2007; Davies and Vethaak 2012).

Biological responses measured at individual, cellular, and subcellular level are commonly referred to as biomarkers. As demonstrated in numerous studies, biomarkers have diagnostic power with regard to both exposure to and effects of contaminants (Broeg et al. 1999; Sturve et al. 2005; Lang et al. 2006). Integrated indices and similar approaches (expert systems, environmental prognostics, etc.) based on the measurement of a set of biomarkers have recently been developed and tested in the field. Application of these methods allows for comparisons of the “health status” of populations (i.e., an integrated estimate of the performance capability of a population based on measurements of potential dysfunctions or damage at different biological levels, a definition applied for the purpose of this study) inhabiting different locations. Furthermore, a mathematically derived integrated index consisting of chemical, biomarker, and population/community data can facilitate an overall assessment of the ecosystem health of an area, which is obviously also affected by eutrophication, habitat disturbance, overfishing, noise, etc. Such indices have also been shown to be useful to communicate the results of complex interactions to environmental managers and the public (ICES 2007).

The Baltic Sea suffers from a multitude of stressors generated by human activities. Besides the steadily progressing eutrophication and related problems, the threat of ecological effects caused by hazardous substances has not receded, despite the reduced environmental concentrations of some “classical” contaminants. In fact, the profile of chemical exposure has shifted to include a much wider range of different types of compounds used by humankind for various purposes (consumer chemicals, pharmaceuticals, plastic additives, surfactants, etc.). This has led to a situation where there is uncertainty of the true environmental risk posed by these new chemical mixtures with steadily elevating concentrations in the marine environment, and in terms of how to detect and assess the significance of the problem. The necessity to include biological effects methods in the monitoring and assessment toolbox has therefore gained weight markedly.

The EU 5FP BEEP project (Biological Effects of Environmental Pollution in Marine Coastal Ecosystems, 2001–2004) was the first extensive joint effort for the development of tools and approaches for monitoring the biological effects in the Baltic Sea area (Lehtonen and Schiedek 2006). Requirements for an integrated monitoring program were identified and considered in relation to the assessment of ecosystem health (Lehtonen et al. 2006). The next major international collaboration project BEAST (Biological Effects of Anthropogenic Chemical Stress: Tools for the Assessment of Ecosystem Health) executed under the Baltic Sea BONUS program continued on the main tracks of BEEP by testing and validating the biomarker methodology. However, the focus was now increasingly directed to the development of integrated assessment of chemical pollution and tools contributing to the holistic ecosystem health assessments (Table 1).

**Table 1:** The BEAST project: main goals and outputs

Goals	Outputs	Related publications
Research, application, and evaluation of established and new biomarker techniques with special focus on biological effects of selected important chemical compound groups in Baltic Sea key species in the laboratory and under field conditions	14 sampling campaigns covering five areas (Belt Sea, Gulf of Gdansk, G. of Riga, G. of Finland, and G. of Bothnia); a total of 20 different biological effects methods tested on 16 species (when feasible); specific laboratory exposure experiments and sediment bioassays	Subregional assessment articles 2014, in prep.; Dabrowska et al. (2012, 2013), Kreitsberg et al. (2012), Höher et al. (2012), Fricke et al. (2012), Baršienė et al. (2012), and Berezina et al. (2013)
Scientifically based recommendations for the set-up of an integrated chemical–biological effects monitoring of hazardous substances in the whole Baltic Sea area, based on subregional assessments for future integrated assessments of Baltic Sea ecosystem health	See above (subregional assessments; collaboration with ICES SGEH and HELCOM)	ICES (2011a) and HELCOM (2012a, b)
Generation of baseline data for regions in the Baltic Sea where few or no biological effects data existed and updating of data in other subregions	See above; BonusHAZ database (jointly with BALCOFISH project) with 60 different parameters for fish (flounder, eelpout, and herring) and invertebrates (*Mytilus* sp., *Macoma balthica*, amphipods, and gastropods)	Subregional assessment articles 2014, in prep.; Barda et al. (2013), Fricke et al. (2012) Baršienė et al. (2012) and Turja et al. (in press)
Identification of relevant target species for the highly variable Baltic Sea subregions	Testing of local native organisms for assessing the suitability of biological effects methods	Subregional assessment articles 2014, in prep.; Barda et al. (2013)
Determination of subregional reference/target/effect levels and collection of data for whole-region assessment of biological effects	Selection of CORESET biological effects indicators for hazardous substances (lysosomal membrane stability in fish, bivalves, or amphipods; induction of micronuclei in fish, bivalves, or amphipods; embryo aberrations in fish [eelpout] or amphipods; Fish Disease Index; imposex in marine gastropods (TBT indicator); PAH metabolites in fish [PAH indicator]); Numeric Assessment Criteria for Baltic Sea organisms to assess biological effects; overview of Core and selected Candidate Indicators to assess the effects of hazardous substances at different biological levels; ICES SGEH Biological Effects methods’ Background Documents for the Baltic Sea region (9 methods)	ICES (2011a), HELCOM (2012a, b) and Baršienė et al. (2012)
Linking early effects and higher level effects by relating responses directly to changes in growth, reproductive output, or energy utilization	Experimental and field studies with mussels (*Mytilus trossulus*) and amphipods (*Monoporeia affinis*)	Turja et al. (in press) and Löf et al. (in prep.)
Subregional health assessments by the application of techniques representing various biological processes at different levels of biological organization in combination with contaminant measurements in different subregions of the Baltic Sea	See above; analysis of PAHs, trace metals, organotins, and organochlorine compounds in sediments, clams, and fish from different subregions	Subregional assessment articles 2014, in prep.; Dabrowska et al. (2012, 2013)
Testing and validation of integrated monitoring approaches, indices, and expert systems with regard to their applicability for the Baltic Sea, taking into account the specific biotic and abiotic characteristics of the different subregions and different contaminant burdens	See above; mussel caging studies in G. of Gdansk and G. of Bothnia; testing of integrated indices (IBR, IBAS) in fish (herring and eelpout); testing of an expert system on amphipods (*Monoporeia affinis*); application of the Fish Disease Index	This article; subregional assessment articles 2014, in prep.; Dabrowska et al. (2012, 2013), Fricke et al. (2012) and Turja et al. (in press)
Cooperation with the expert groups of ICES and HELCOM for providing recommendations to the ongoing revision of HELCOM monitoring programs, and implementation of the BSAP and MSFD	Close collaboration with HELCOM CORESET; exchange of information between relevant ICES expert groups; support to BSAP and MSFD at national levels	ICES (2011a, b, 2012) and HELCOM (2012a, b)
An integrated multilevel toolbox consisting of established and novel biomarkers as sensitive diagnostic tools to identify how hazardous substances affect the Baltic Sea ecosystem, also in the context of stress due to varying environmental conditions and climate change	See above (subregional assessments, selection of methods, and determination of assessment criteria); experimental studies on mussels and fish	Subregional assessment articles 2014, in prep.; Dabrowska et al. (2012, 2013), Höher et al. (2012) and Gorokhova et al. (2013)
Capacity building and strengthening of networking and quality assurance among Baltic Sea institutions via workshops to exchange, harmonize, and intercalibrate methodologies; development of technical guidelines and Standard operation procedures as well as appropriate training	Intercalibration and workshops; draft Standard operation procedures, training; networking activities	Kammann et al. (2013) and Standard operation procedures (2014, in prep.)

## Basic Research: Development of New Methods and Testing of Potential Target Species in Different Subregions of the Baltic Sea

The basic research component of BEAST focused mainly on field studies regarding biological effects of hazardous substances. Measurements were made at various levels of biological organization in local target organisms considered promising for environmental monitoring purposes. Fourteen field campaigns were performed during 2009–2011 in the Belt Sea, Gulf of Gdansk, G. of Riga, G. of Finland, and G. of Bothnia subregions. Key species representing algae, zooplanktonic and benthic crustaceans, bivalves, gastropods, and fish were collected and analyzed for a wide range of biological effects by applying a variety of novel (Table 2) and established methods. Bioassays using crustacean amphipod species and sediments collected from the subregions as well as analyses of selected chemical contaminants such as polycyclic aromatic hydrocarbons (PAH), organochlorines, organotins, and trace metals from sediments and/or tissues were performed to assess the degree of contamination of the studied areas. Field transplantation studies using caged blue mussels were carried out in selected coastal areas. In addition to the field studies, a range of specific experimental studies were carried out to investigate combined effects of contaminants and other environmental stressors including hypoxia, salinity, pH, and eutrophication.

**Table 2:** Use of novel methods or established methods applied on new species relevant for the Baltic Sea

Method name	Short study description	Species	Study areas	References
Oxidative capacity in amphipods	Antioxidant status in amphipods from contaminated sediments using the oxygen radical capacity (ORAC) assay was tested for different ontogenetic stages (embryos, juveniles, and gravid females) and applied for several amphipod species. For validation, ORAC was measured together with the antioxidant defense enzyme superoxide dismutase (SOD) and catalase (CAT) in *M. affinis* exposed to contaminated sediment and hypoxia	*Monoporeia affinis,* *Gammarus* *tigrinus,* and *Gammarus zaddachi*	G. of Bothnia, G. of Riga, and G. of Gdansk	Löf et al. (in prep.)
Acetylcholinesterase activity in amphipods	Neurotoxic effects were studied with the biomarker acetylcholinesterase activity in amphipods	*Monoporeia affinis,* *Gammarus* *tigrinus*, and *Gammarus zaddachi*	G. of Bothnia, G. of Riga, and G. of Gdansk	Löf et al. (in prep.)
Lysosomal membrane stability in amphipods	Lysosomal membrane stability using the fluorescent dye acridine orange (AO) as a subcellular biomarker indicating general health status of organism was studied in amphipods	*Monoporeia affinis*	G. of Bothnia	Broeg et al. (in prep.)
Intersex in amphipods	Intersex, an indicator of endocrine disruption because of the presence of both female and male sex characteristics (i.e., male-like penile papillae and female-like oostegites without setae) in the same individual was studied in two genera of amphipods from the Belt Sea.	*Gammarus* sp, *Corophium volutator*	Belt Sea	Fisher and Strand (unpublished)
Reproductive success in amphipods	Embryo aberrations as a general biomarker for pollution effects were studied in amphipods from coastal zones by adjusting the method for reproduction success developed earlier for *M. affinis* (Sundelin et al. 2008)	*Gammarus* *tigrinus*, *Gammarus zaddachi*, *Corophium volutator*	G. of Bothnia, G. of Finland, G. of Riga, G. of Gdansk, Belt Sea	Sundelin et al. (unpublished) and Fisher and Strand (unpublished)
Cardiac activity in crabs and mussels	Recovery time of heart rate after standardized test-stimulus, i.e., experimentally lowered salinity or temperature, was used as an integrated physiological biomarker of general health in organisms for studying pollution effects	*Carcinus maenas*, *Mytilus edulis*	Belt Sea	Kholodkevich et al. (in prep.)
Oxidative stress in clams	Activity of the antioxidant defense enzyme glutathione reductase (GR) was studied in combination with other biomarkers in soft-bottom clams collected from most of the BEAST subregions, with additional studies on seasonal variation	*Macoma balthica*	G. of Bothnia, G. of Finland, G. of Riga, and G. of Gdansk, Belt Sea	Barda et al. (2013)
Cellular immune responses in mussels	Biomarkers for immune responses (i.e., total and differential haemocyte count, phagocytic activity and apoptosis) were studied in mussels from areas characterized by variable salinities and different contaminant profiles	*Mytilus edulis*	Belt Sea	Höher et al. (2012)
Micronuclei frequency in amphipods and fish	Micronuclei and other nuclear abnormalities related to geno- and cytotoxic effects were studied in amphipods, eelpout and herring	*Gammarus locusta*, *Zoarces viviparus*, *Clupea harengus*	G. of Finland, G. of Bothnia, G. of Riga, and G. of Gdansk, Belt Sea	Baršienė et al. (2012) and Baršienė et al. (unpublished)
Oil-degrading bacteria in the intestinal tract in bivalves and fish	The presence of oil-degrading bacteria in the intestinal tract was studied in bivalves and fish as a new method for in situ assessment of petroleum hydrocarbon pollution in marine and estuarine environments	*Mytilus trossulus*, *Macoma balthica*, *Zoarces viviparus*, *Platichthys flesus*, *Clupea harengus*	Different subregions of the Baltic Sea	Baršienė et al. (unpublished; in press)
Liver histopathology in fish	Liver lesions were studied in eelpout and herring as the first attempt to carry out systematic studies on these species by analyzing samples from all studied subregions by applying diagnostic criteria originally developed for flatfish species (Feist et al. 2004; Lang et al. 2006)	*Zoarces viviparus*, and *Clupea harengus*	G. of Finland, G. of Bothnia, G. of Riga, and G. of Gdansk Belt Sea	Fricke et al. (2012), Fricke et al. (in prep.) and Gschwind (2012)
Macrophage aggregates in fish	Macrophage aggregates were quantitatively studied in fish liver and spleen	*Zoarces viviparus*, and *Platichthys flesus*	G. of Finland, G. of Bothnia, G. of Riga, and G. of Gdansk Belt Sea	Fricke et al. (2012) and Dabrowska et al. (2012)
Intersex in fish	Intersex, regarded as the consequence of an endocrine disruption effects in gonads of male fish, was studied by histological analysis	*Zoarces viviparus*, and *Platichthys flesus*	G. of Finland, G. of Bothnia, G. of Riga, G. of Gdansk, and Belt Sea	Gercken et al. (in prep.)
PAH metabolites in fish urine	PAH metabolites in fish urine as a biomarker of PAH-exposure was studied in addition to PAH metabolites in bile fluid	*Zoarces viviparus*, and *Platichthys flesus*	G. of Riga, Belt Sea	Kreitsberg et al. (2012) and Tairova et al. (2012)
Oxidative stress in macroalgae	Oxidative stress biomarkers were studied by measuring GST and GR in bladder wrack from different areas influenced by riverine input and pollution	*Fucus vesiculosus*	G. of Riga, different subregions of the Baltic Sea	Boikova (unpublished)

Many of the biological effects methods applied in this project have been recommended by, for example, expert groups of the International Council for the Exploration of the Sea (ICES) and The Convention for the Protection of the Marine Environment of the North-East Atlantic (OSPAR) to be applied in the integrated monitoring of contaminants (ICES 2007, 2011b; Davies and Vethaak 2012). However, they were still considered to be in need for more research, e.g., for the establishment of assessment criteria (background and threshold values) in different species. Besides the effects of contaminants, there was a quest for the examination of possible impacts of salinity and other environmental variables on the responses. Some of these methods were eventually selected to compose the set of methods recommended for the CORESET project of the Helsinki Commission for the protection of the Baltic Sea (HELCOM) for the strengthening of biological effects monitoring that has been running clearly behind the progress going on in other sea areas of Europe (Lehtonen and Schiedek 2006; Lehtonen et al. 2006; HELCOM 2010).

## Development of Monitoring: Database, Selection of Endpoints, and Integrated Assessment

BEAST worked in close collaboration with HELCOM CORESET (HELCOM 2012a, b) and the ICES Study Group for the Development of Integrated Monitoring and Assessment of Ecosystem Health in the Baltic Sea (SGEH) to develop the application of bioeffect tools in the monitoring and assessment of impact of anthropogenic contaminants on the Baltic Sea ecosystem. This resulted in the preparation of scientific background documents, including Baltic Sea specific assessment criteria for different biomonitoring species and biological effects, and recommendations for biological effects methods to be used as core and candidate indicators for the revised monitoring program of the Baltic Sea and the implementation of the EU Marine Strategy Framework Directive (MSFD) (HELCOM 2012a, b). The main achievements included also the construction of the database “BonusHAZ,” jointly with the BONUS BALCOFISH project, and exploring of tools for integrated assessment between contaminant levels, different biomarkers, and/or species.

### Database

The “BonusHAZ” database forms, to date, the most comprehensive data collection on biological effects of hazardous substances in the Baltic Sea region, consist of 60 different parameters for more than 600 single specimens of different fish and invertebrate species in different subregions of the Baltic Sea. As of 2010, BEAST has a formal arrangement with ICES to use their database code lists (RECO), and the “BonusHAZ” is planned to become available in public domain at a later stage.

### Core Methods and Assessment Criteria

With the new database and along with information from various national initiatives and monitoring activities in the Baltic Sea countries as well as improved scientific understanding of cause-and-effect relationships, sufficient data are now available and used to develop Baltic Sea-specific assessment criteria for biological effects of contaminants. In fact, this is a prerequisite for including any method in a monitoring program. A set of specific biological effects indicators tailored for the Baltic Sea has now been selected, based on knowledge obtained during scientific research in the area, e.g., within the BEEP, BEAST, and BALCOFISH projects, as well as from the work performed in relevant ICES and OSPAR expert groups.

The chosen core indicators listed below describe either effects caused by mixtures of contaminants or specific contaminants. They also indicate adverse effects at different biological levels, i.e., molecular/biochemical/cellular (“early warning”) or individual/population (health and reproductive impairments) levels. For these indicators, cause–effect relationships have been studied, and assessment criteria have been established. Furthermore, being important for method of harmonization across the sea areas of Europe, these are the established methods recommended by ICES expert groups, and many have already been included in other regional monitoring programs, e.g., under OSPAR and MEDPOL, or in national monitoring programs (Denmark, Sweden, and/or Germany). The recommended core methods are:

- General stress caused by a range of contaminants (“early warning”): lysosomal membrane stability in fish, bivalves, or amphipods;
- Effects caused by genotoxic contaminants (“early warning”): induction of micronuclei in fish, bivalves, or amphipods;
- Reproductive success impairments caused by a range of contaminants: embryo aberrations in fish (eelpout) or amphipods;
- General health status: Fish Disease Index based on externally visible fish diseases, macroscopic liver neoplasms, and liver histopathology.

In addition, two contaminant-specific biological effects indicators, imposex in marine prosobranch gastropods and PAH metabolites in fish, have been included as part of the core indicators for TBT and PAH, respectively. Finally, a few methods have been placed in the candidate list, including intersex or vitellogenin induction in male fish (endocrine disruption), acetylcholinesterase activity (neurotoxicity), and ethoxyresorufin-*O*-deethylase activity (biotransformation).

The future application of biological effects indicators in a combined monitoring strategy strengthens the assessment of contaminants that are approaching critical concentration levels giving rise to pollution effects in the Baltic Sea. Inclusion of biological effects indicators into the HELCOM Cooperative Monitoring in the Baltic Marine Environment (COMBINE) program in the future also allows for the detection of combined effects of complex mixtures of hazardous substances, which is the norm for most sea areas. In using only contaminant-specific effect methods, such as imposex in prosobranch gastropods or PAH metabolites in fish, a much larger number of indicators would have to be applied to cover the effects of all relevant groups of hazardous substances present in the marine environment. In addition, most of them would still be neglected since some of the process-related chemicals released to the Baltic Sea are presently unidentified. Effect indicators with a wider detection spectrum pinpoint the need for further investigations to identify the chemicals causing the effects observed. Besides fulfilling the requirement of the Baltic Sea Action Plan (BSAP), inclusion of core indicators of biological effects into the HELCOM COMBINE program will be an important step toward harmonization of environmental assessments of different sea areas in Europe; eventually, this will allow for comparisons on larger scales and, thus, implementing important main goals of the MSFD.

### Testing of Integrated Approaches

At present, various methodologies are either available or under development to monitor and assess pollution effects and ecosystem health in marine and coastal waters. Integrated indices and similar approaches (e.g., multivariate approaches, expert systems, and decision support systems) based on the measurement of a set of biomarkers have recently been developed and applied in the North Sea, the Northeast Atlantic, and the Mediterranean Sea (Cajaraville et al. 2000; Beliaeff and Burgeot 2002; Moore et al. 2004; Broeg and Lehtonen 2006; Dagnino et al. 2007; Viarengo et al. 2007; Hagger et al. 2009; Marigómez et al. 2013). During BEAST, feasibility of application of these approaches for integrated assessment between contaminant levels, different biomarkers, and/or species was explored (Höher et al. 2012; Dabrowska et al. 2012, 2013; Turja et al. in press). These studies showed different patterns in biological responses between the more- and less-contaminated sites, often also with discrimination between effects caused by contaminants and salinity.

One example of the data integration methods was the application of the Integrated Biomarker Assessment Tool (IBAT), developed jointly by BEAST and BALCOFISH, and tested on eelpout (Strand et al. unpublished data). The IBAT tool allows for comparisons of input data for the measured biological effect parameters for which assessment criteria have been developed, and an overall Integrated Biomarker Assessment Score (IBAS) can thus be calculated. IBAS includes weighted score values depending on the biological response level of the respective effects, and therefore, considers the biological significance of the effects observed, i.e., subcellular (Score 1), cellular/tissue (Score 2), or whole organism responses (Score 3). IBAS summarizes all weighted scores by the formula IBAS = (Σ *X*)/(sqrt *n*), which follows the principles of the indicator-based integrative assessment tool CHASE, developed during the HELCOM Integrated Thematic Assessment of Hazardous Substances in the Baltic Sea (HELCOM 2010). CHASE has been used to integrate the status of contamination by individual chemicals and biological effects at specific sites or areas into a single status value termed “contamination ratio.” The assessment of the IBAS value follows the “traffic light” assessment scale as applied in the CHASE tool, i.e., <1 (low impact), 1 to <5 (moderate impact), and >5 (high impact). Subsequently, IBAT has the potential to be incorporated as an improvement into the CHASE tool for future assessments. IBAS also has the potential to be used for a single-species health index if enough data for relevant biological effect indicators are available, or for several species as an integrative measure for the all the observed biological effects in a particular area.

**Fig. 1:**
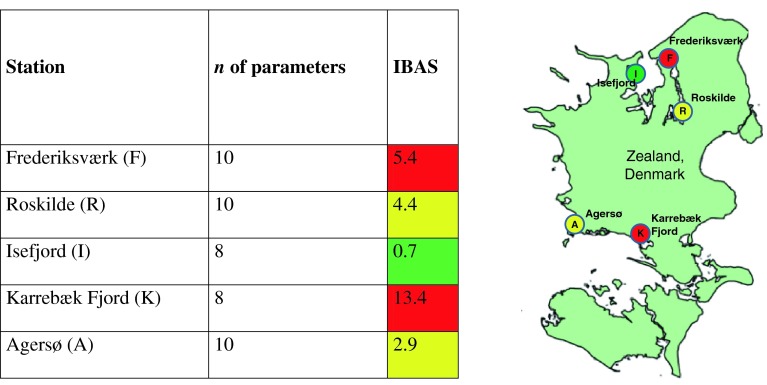
IBAS with the subsequent “traffic-light” assessment for five eelpout (*Zoarces viviparus*) sampling stations in the Danish part of Belt Sea (Strand et al. in prep.)

An example of the use of IBAT is given in Fig. 1, showing the outcome of a study on eelpout in selected coastal areas in Denmark. The IBAS indicates a relatively high impact of pollution in eelpout at two of the stations investigated, whereas at two others, the impact is moderate. Only at one station, a low impact of pollution is indicated. Considering the available information on contaminant levels at the respective stations, the impact levels of pollution in eelpout defined by IBAT are considered feasible.

The integration of several effect biomarkers measured at different levels of biological organization up to the level of reproduction has been a further challenge with respect to incorporating all information into an integrated assessment and to provide a science-based “traffic light” score. Thus, IBAT has been developed based on data from all biological effects indicators, and it can be used to perform an overall assessment of risk level for pollution effects at a specific location. The application of the integrated pollution–response model and the use of a decision support system for the ranking of responses from the cellular to the reproduction level in amphipods have proven to be promising approaches (Löf et al. in prep.).

**Fig. 2:**
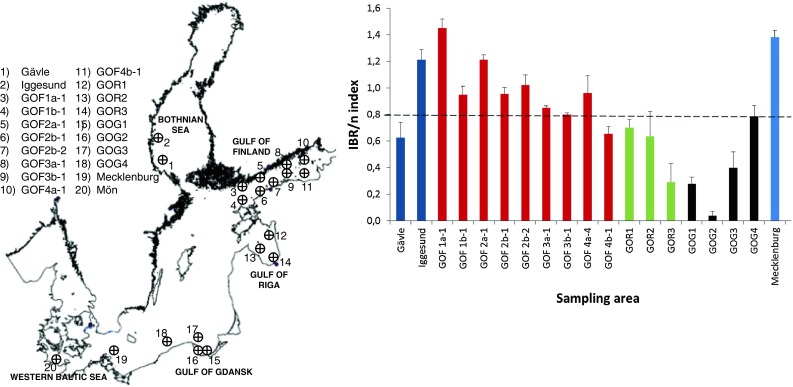
Integrated Biomarker Index (IBR/*n*, mean ± SE for different parameter orders) in Baltic herring (*Clupea harengus membras*), calculated for female individuals collected during BEAST cruises of r/v *Walther Herwig III* in December 2009 and 2010, except in the Gulf of Finland (August 2009). Biomarkers (5) used for the index: catalase activity (oxidative stress); acetylcholinesterase activity (neurotoxicity), glutathione *S*-transferase activity (biotransformation phase II); lysosomal membrane stability (general stress); and histopathology (general health). The *dashed line* indicates the mean index value for this data

Another example of integrated approaches performed under BEAST is given in Fig. 2. Biological effects data obtained from females of herring collected from all the studied subregions were fed into a simple algorithm set-up, the Integrated Biomarker Response (IBR) (Beliaeff and Burgeot 2002; Broeg and Lehtonen 2006). The results of the IBR approach demonstrate the value of integrated methods in describing the combined environmental stress experienced by organisms at different field sites in relation to each other.

## Operational and Practical Issues in Ecosystem Health Assessments: Sampling, Intercalibration, and Quality Assurance

In addition to basic scientific research and method testing, the BEAST project set out to prepare methodological guidelines and standard operating procedures for relevant biological effects techniques, and, in association with that, to organize and execute training and intercalibration workshops and activities.

### Guidelines and Standard Operating Procedures

The implementation of guidelines and standard operating procedures is a fundamental quality assurance requirement for monitoring and assessment. Although guidelines for many biological effects techniques already exist (largely developed by ICES), they often lack coherence and do not sufficiently address the specific environmental conditions of the Baltic Sea. Therefore, it was necessary to develop specific guidelines and standard operating procedures for those biological effects techniques identified by BEAST as being useful for the Baltic Sea. Guidelines and instructions generated were subsequently transferred to draft standard operating procedures, focusing on the core and candidate indicators identified by HELCOM CORESET (Table 3). These standard operating procedures will be published once the results of the present revision of HELCOM’s monitoring program with regard to indicators for future monitoring and the structure of the monitoring guidelines become available. It is envisaged that standard operating procedures for other techniques applied and assessed in the BEAST project will be developed at a later stage. Further research is needed to integrate the BEAST draft standard operating procedures with those on chemical measurements that are partly existing (e.g., under HELCOM COMBINE) or are currently under development. The preparation of the guidelines and standard operating procedures can be considered as a major milestone for the implementation of a quality-assured integrated monitoring and assessment program for the Baltic Sea that will meet the goals and ecological objectives of HELCOM BSAP and EU MSFD in relation to hazardous substances.

**Table 3:** Draft BEAST standard operating procedures based on HELCOM CORESET requirements regarding “core” and “candidate” indicators (intended to be published as HELCOM documents after revision of the HELCOM monitoring program)

Indicator type	Title of SOP	Authors
General	Sampling for monitoring biological effects of contaminants in the Baltic Sea	Lang T., Lehtonen K., Sundelin B., and Schiedek D.
CORESET Core Indicator (Bioeffects)	Reproductive success in fish	Strand J. and Gercken J.
	Reproductive success in amphipods	Sundelin, B.
	Micronucleus test in fish and bivalves	Baršienė J.
	Lysosomal membrane stability	Broeg K., Schatz S., Strand J., and Lehtonen K.
	Fish disease monitoring in the Baltic Sea. Part A: Externally visible diseases	Lang T., Rodjuk G., and Fricke N.
	Fish disease monitoring in the Baltic Sea. Part B: Macroscopic liver neoplasms	Lang T., Fricke N., Rodjuk G., and Dabrowska H.
	Fish disease monitoring in the Baltic Sea. Part C: Liver histopathology	Lang T., Fricke N., Rodjuk G., and Dabrowska H.
CORESET Core Indicator (PAH)	Determination of PAH metabolites in fish bile	Kammann U.
CORESET Core Indicator (TBT)	Imposex in marine snails	Strand J. and Gercken J.
CORESET Candidate Indicator (Bioeffects)	Intersex (ovotestis) measurement in eelpout (*Zoarces viviparus*)	Gercken J.
	Measurement of vitellogenin in the blood plasma of fish	Fricke N.
	Determination of acetylcholinesterase activity in fish and bivalves	Lehtonen K. and Gercken J.
	Determination of EROD activity in fish	Vuorinen P., Tuvikene A., Dabrowska H., and Lang T.

### Training and Intercalibration

Training of personnel and intercalibration of methodologies applied in research and monitoring are essential for their implementation in joint monitoring and assessment programs. Together with the establishment and use of guidelines and standard operating procedures they form the essential components of quality assurance. The importance of quality assurance, especially in international monitoring programs with contributions from several national institutes/laboratories into a common data pool, has been clearly recognized.

Aspects covered during BEAST intercalibration and training exercises included sampling of biotic and abiotic matrices, processing and conservation of samples, on board examination of the health status of organisms, laboratory-based analysis of samples, and data treatment (Table 4). While intercalibration was mainly meant as an internal process (involving BEAST, and, partly, BALCOFISH partners) to improve the comparability of results, many of the workshops arranged were open to both project partners and external interested colleagues, e.g., the intercalibration of PAH metabolite measurement in fish bile attracted several non-BEAST specialist laboratories from the OSPAR area (Kammann et al. 2013).

**Table 4:** BEAST training and intercalibration activities

Title of activity/objectives	BEAST Lead Laboratory	Venue	Results
Training in analyses of methods in amphipod reproduction, embryo aberrations, sperm count, and intersex	University of Stockholm, ITM (SE)	Stockholm University Marine Science Center, Askö Laboratory (SE)	Methods for the analysis of reproductive disorders in amphipods were presented and trained
Intercalibration on imposex and intersex in marine gastropods	QUASIMEME	Samples sent for analysis	Satisfactory *Z*-scores between −0.6 and 0.4 were achieved for the reported three parameters by the participating BEAST laboratory
Training and intercalibration of field sampling for integrated studies on contaminants and biological effects	TI Institute of Fisheries Ecology (DE); SYKE (FI)	Onboard RVs “*Walther Herwig* III” (DE) and “*Aranda*” (FI)	Strategies and concepts for integrated monitoring and assessment of hazardous substances were presented, and various methods relevant for the BEAST project and for integrated monitoring (chemistry, biomarkers, and bioassays) in the Baltic Sea in general were demonstrated by instructors and trained by the participants
Joint BALCOFISH/BEAST practical workshop on eelpout sampling and examinations	Aarhus University, NERI (DK)	Søminestationen, Holbæk (DK)	A practical workshop with 13 BALCOFISH and five BEAST partners. Issues addressed included standardization of methodologies for sampling and dissection eelpout, assessing reproductive success, and reporting of data to the common databank BonusHAZ
Workshop on measurement of enzymatic biomarkers in bivalves	SYKE (FI)	SYKE, Marine Research Laboratory, Helsinki (FI)	Dissemination of biomarker methods to Latvian partners and intercalibration of methods
Training and intercalibration exercise of the histochemical method for the assessment of lysosomal membrane stability	AWI (DE)	Alfred Wegener Institute for Polar and Marine Research, Bremerhaven (DE)	LMS in herring samples was successfully analyzed and assessed at TI. Intercalibration showed a very high correspondence of results
PAH metabolite intercalibration exercise	TI Institute of Fisheries Ecology (DE)	Samples were distributed to participating labs	The relation of the five concentration levels of 1-hydroxypyrene could be detected by all labs except one. So, all methods are in general suitable for screening purposes. HPLC-F and GC–MS produced quite similar results in absolute concentration. SF results were treated with a conversion factor and tended to be higher than HPLC-F and GC–MS but were not significantly different. The concentrations determined with FWF were not comparable to those from the other methods and were in addition inhomogeneous within the method (more than 10-fold difference)
Biomarkers – effects of hazardous substances in aquatic ecosystems (seminar)	SYKE (FI)	SYKE, Helsinki (FI)	The event was the first large seminar arranged in Finland focusing on the use of biological effects methods in marine monitoring and assessment of hazardous substances. More than two-thirds of the seminar audience consisted of representatives of national and municipal environmental monitoring authorities, industry, SMEs, NGOs, and educational institutes, with the rest being researchers and students
Liver histopathology in eelpout	TI Institute of Fisheries Ecology (DE)	TI Institute of Fisheries Ecology, Cuxhaven (DE)	Liver histopathology in eelpout was presented, and methods relevant for the BEAST project were demonstrated by the instructors and were trained by the participants

Overall, the BEAST training/intercalibration activities contributed to the enhancing of quality assurance, capacity building, and networking in the Baltic Sea region. It is emphasized that internal and external training and intercalibration have to be understood as dynamic processes that need to be carried out on a continuous basis as an important part of marine monitoring and assessment. It is, thus, essential that the revised HELCOM COMBINE program (as well as the future monitoring under the MSFD) encompasses an operative quality assurance component.

## Conclusions and Future Challenges

The research activities carried out in the BEAST project were focused on the further development of existing biological effects techniques to be applied in Baltic Sea subregions. The results show that hazardous substances are currently causing a wide variety of biological responses in the key species selected and must therefore be regarded as a continuous threat to the health of the Baltic Sea ecosystem. Based on these results, BEAST identified a set of core and candidate biological effects techniques (indicators) for future monitoring of hazardous substances and their effects, and developed and tested integrated assessment strategies, including numeric criteria for the assessment of the environmental status of the Baltic Sea. Furthermore, an extensive database, comprising conceptual and methodological background documents as well as quality assurance components, was developed, all of which are required in the implementation processes concerning integrated monitoring at the national and international levels. Recommendations made by BEAST were taken up by the HELCOM CORESET project and international expert fora, and their implementation in respect of national monitoring is under discussion at present. However, despite the progress achieved, additional work still remains to be done:

- Some biological effects techniques regarded as promising (e.g., the candidate indicators above) still need to be further developed and validated.
- A stronger linkage between measurements of contaminants and biological effects at various levels of biological organization needs to be achieved to facilitate integrated monitoring and assessment. This will require new approaches, addressing, e.g., source identification, means of discharge to the sea, chemical behavior in the environment, biotic pathways and responses (biological degradation, biomagnification, bioaccumulation, and toxicity), and fate (chemical degradation, deposition).
- An improvement of current assessment concepts and criteria is required to be able to tackle ecologically relevant problems such as toxicity of chemical mixtures, multiple stress, and multilevel effects. This can be reached by applying and evaluating new methods and model approaches (e.g., passive sampling, integrated biological effects methods, distribution and fate models, decision trees, substance flow analyses, etc.) and establishing cause–effect links between different levels of biological organization by dedicated laboratory and field studies with ecologically relevant target species. Integrated indicators and indices as well as AC should still be developed.
- The assessment and prediction of ecological and socioeconomic consequence of hazardous substances should be improved. Databases and models from BEAST and earlier projects can be exploited to create scenarios for biological effects. These will supply important information for socioeconomic analyses of changes, e.g., in fish populations, remediation measures of contaminated sediment and waste water-treatment plant effluents, and relevance for human food quality. Evaluations of the cost–efficiency of different monitoring and assessment methods, and the benefits of policy implementation and abatement strategies, as well as exploration of the efficiency of reduction measures with regard to impacts observed in the marine environment are urgent tasks.

Finally, although the BEAST project contributed to the international networking of institutions in charge of monitoring and assessment of hazardous substances in the Baltic Sea, there is still a requirement for enhanced international coordination and harmonization; especially in the light of the requirements of the EU MSFD concerning regional cooperation, this will be a challenging task to accomplish in the coming years.
